# Environmental Risk Assessment of Metals in Groundwater in an Area of Jiujiang City, Jiangxi Province, China

**DOI:** 10.3390/toxics13030197

**Published:** 2025-03-10

**Authors:** Minghao Tian, Shihan Xue, Fujiang Hui, Weiyuan Cao, Ping Zhang

**Affiliations:** 1 Guangxi Key Laboratory of Theory and Technology for Environmental Pollution Control, Guilin University of Technology, Guilin 541006, China; 6616029@glut.edu.cn (M.T.); 1814730343@163.com (S.X.); h19507737192@163.com (F.H.); 2 Engineering Research Center of Watershed Protection and Green Development, Guilin University of Technology, Guilin 541006, China; 3 Guangxi Engineering Research Center of Comprehensive Treatment for Agricultural Non-Point Source Pollution, Guilin University of Technology, Guilin 541006, China; 4 Modern Industry College of Ecology and Environmental Protection, Guilin University of Technology, Guilin 541006, China; 5 Collaborative Innovation Center for Water Pollution Control and Water Safety in Karst Area, Guilin University of Technology, Guilin 541006, China

**Keywords:** metals, groundwater, environmental risk assessment, non-pollution level

## Abstract

To conduct an environmental risk assessment for metals in the groundwater of a site in Jiujiang City, Jiangxi Province, we analyzed seven metals (Cr, Zn, Pb, Ni, Sb, Cu, and Tl) that exhibited higher detection rates among the elements we measured. For example, in our measurement data, the average concentration of the element cobalt (Co) is less than 2 × 10^–3^ μg/L, and the average concentration of the element cadmium (Cd) is less than 5 × 10^–3^ μg/L. The purpose of this environmental risk assessment was to provide a scientific basis for site remediation and subsequent construction. The risk assessment was carried out using the single-factor pollution index, the Nemerow comprehensive pollution index (Pn), and potential ecological hazard index methods. Principal component analysis and correlation analyses were used to investigate the sources of metal pollution in the groundwater. The results indicated the following: (1) The average concentrations of the seven metals in the groundwater of the study area did not exceed the Class IV groundwater quality standard limits. The highest average concentration was for Zn (38.08 μg/L), indicating that metal concentrations in the groundwater were relatively low. (2) The Pn for the seven metals was below 0.7, indicating that the study area was at a non-polluted level. (3) The correlation and principal component analyses of the metals indicate that the sources of these metals may be residues from material stored in the raw material warehouse of the former iron smelting plant at the site. The results show that the level of groundwater contamination at the site falls within an extremely low range; thus, the focus on groundwater pollution can be reduced in subsequent site remediation and construction activities.

## 1. Introduction

Groundwater resources account for a third of China’s total water resources and approximately 20% of the country’s total water supply, providing high-quality freshwater resources for human production activities [1,2,3]. However, with the rapid development of China’s social economy and accelerated industrialization and urbanization, metal pollution in groundwater has become increasingly problematic [4,5,6]. Metal pollutants mainly enter water bodies through industrial discharge or domestic sewage, the release of metals accumulated in river sediments back into the water column, and atmospheric deposition. Because of their toxicity, high concealment, strong persistence, and low degradation, once groundwater is polluted by metals, they pose a threat to human health and environmental safety. High concentrations of Cr^3+^ can still have adverse effects on aquatic life and human health, such as causing skin irritation and allergic reactions. High concentrations of Zn^2+^ may lead to gastrointestinal discomfort, nausea, and other effects on the human body. Pb^2+^ is a highly toxic ion that can cause severe damage to the nervous system, kidneys, and cardiovascular system even at low concentrations. Ni^2+^ at high concentrations is harmful to aquatic life and human health, potentially causing skin allergies (such as nickel dermatitis), respiratory issues, and the development of certain cancers. Sb^3+^ is a toxic metal ion that can damage the liver, kidneys, and lungs. Prolonged exposure may lead to chronic poisoning, including gastrointestinal discomfort and neurological effects. High concentrations of Cu^2+^ can cause liver and kidney damage, gastrointestinal discomfort, etc. High concentrations of Ti^4+^ may have adverse effects on certain sensitive organisms.

This study focused on the groundwater at a site in Jiujiang City, Jiangxi Province, China; we conducted a systematic environmental risk assessment for seven metals (Cr, Zn, Pb, Ni, Sb, Cu, and Tl) detected at concentrations significantly higher than background levels [7]. Utilizing multiple methods, including the single-factor pollution assessment method, the Nemerow comprehensive pollution index (Pn), principal component analysis (PCA), and correlation analysis, this study provides a detailed evaluation of the pollution characteristics and health risks of these metals in groundwater.

The innovation of this study is reflected in three main aspects: First, addressing the relative lack of research on typical groundwater metal pollution characteristics and environmental risk assessment in China, this study is the first to systematically assess the pollution levels and environmental risks of these seven metals in the groundwater of Jiujiang. Second, by integrating a single-factor pollution assessment method into the comprehensive pollution index method, the study overcomes the limitations of single evaluation methods, providing a more accurate and comprehensive reflection of overall groundwater metal pollution status. Finally, through principal component analysis and correlation analysis, this study explored the source relationships and pollution characteristics among different metals, offering a scientific basis for targeted pollution control strategies [8,9,10].

The practical significance of the research findings lies in providing a scientific basis for site remediation and subsequent construction, as follows: (1) The single-factor pollution index, Nemerow comprehensive pollution index (Pn), and potential ecological hazard index methods, this study identified pollutants that require priority control and their spatial distribution characteristics, providing clear targets for subsequent remediation projects. (2) The pollutant source analysis results offer critical references for developing pollution prevention measures, helping to control pollutant inputs at the source.

From a broader perspective, the outcomes of this study not only provide a scientific basis for addressing specific pollution issues in Jiujiang but also serve as a methodological reference for groundwater metal pollution risk assessment in other similar regions across China [11]. The research results have significant theoretical and practical value for improving China’s groundwater environmental risk assessment system and promoting regional environmental governance. Future research could further incorporate hydrogeological characteristics to explore the migration and transformation patterns of pollutants in aquifers, supporting the development of more scientific pollution remediation plans. Additionally, we recommend establishing long-term monitoring mechanisms to dynamically evaluate pollution control effectiveness, ensuring sustained improvement in groundwater environmental quality [12,13].

## 2. Materials and Methods

### 2.1. Overview of the Study Area

The study area was located in Lianxi District, Jiujiang City, Jiangxi Province (116.0448° E and 29.6924° N). Jiujiang City has a diverse range of groundwater types, including porous water in loose rocks, fracture–karst water in carbonate rocks, and fracture water in the bedrock. Groundwater resources are relatively abundant but unevenly distributed.

### 2.2. Sample Collection and Processing

**Figure 1:**
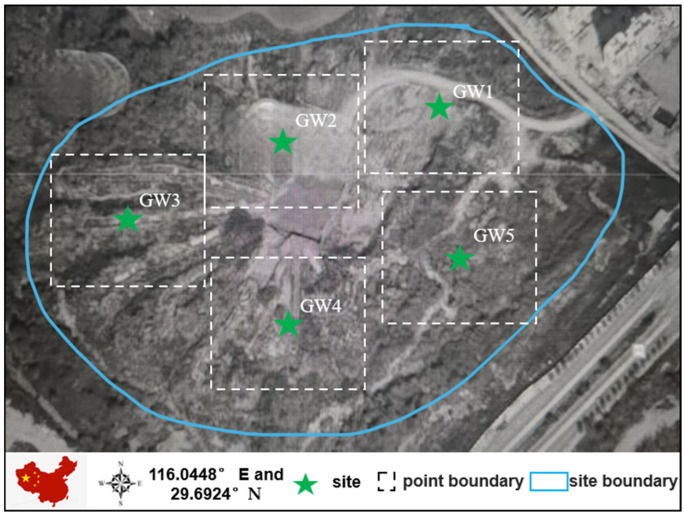
General description of the study area.

Five groundwater survey wells are located at the site. The sampling points were named GW1, GW2, GW3, GW4, and GW5, as shown in Figure 1. The concentrations of seven metals were measured at these points. The sampling was conducted in 2022 on 12 January, 24 February, 15 March, 8 May, 28 May, 30 June, 21 July, 27 October, 17 November, and 17 December. For the ten samplings, the data for each sampling point were evaluated [14]. Sample collection was performed according to standard processes for monitoring logging photography and background material collection, sampling equipment and container preparation, water level and well depth measurement, well washing, on-site monitoring and recording, water sample collection, sample preservative addition for sample preservation, sample inventory, refrigeration, and sample recording.

The groundwater sampling depth was 0.5 m below the well water level. Before sampling, the sampler and sample container were rinsed 2–3 times with the collected water samples. Then, the water sample was filtered into a polyvinyl chloride (PVC) bag using a 0.45 µm filter membrane. The filtered sample was acidified with nitric acid (1 mL of nitric acid per 100 mL of sample) to lower the pH to below 2, thus preventing the precipitation or adsorption of metals. Samples were stored at 4 °C. After being returned to the laboratory, metal concentrations were measured using an inductively coupled plasma mass spectrometer (ICP-MS).

### 2.3. Measurement Methods and Instruments

The seven groundwater metals concentrations of Cr, Zn, Pb, Ni, Sb, Cu, and Tl in this field study were all measured by inductively coupled plasmon mass spectrometry HJ 700-2014, and the instrument used was an inductively coupled plasmon mass spectrometer NexION350D (ICP-MS) (PerkinElmer, Waltham, MA, USA). And the model of the pH meter used was PHB-5.

Instrument Operating Conditions: Instrument Model, NexION 350D ICP-MS (PerkinElmer, USA); RF Power, 1300 W; Nebulizer Gas Flow, 0.98 L/min; Auxiliary Gas Flow, 1.2 L/min; Plasma Gas Flow, 15 L/min; Sampling Cone and Skimmer Cone, Ni; Detection Mode, Standard Mode; Dwell Time, 100 ms; Replicates, 3. The detection limits and quantitation limits for each element analyzed by ICP-MS are shown in Table 1.

We chose the collision mode to set the concentration level of each element’s standard solution and selected the online automatic addition of the internal standard; the concentration of the internal standard working solution was 50 μg/L, and the injection mode was automatic injection. The standard solution was introduced into the ICP-MS injection system in sequence, the ratio of the response values of each element in the standard solution to the response value of the internal standard was taken as the vertical axis, and each concentration was taken as the horizontal axis. A standard curve was drawn for quality control. The working curves and correlation coefficients of the elements are shown in Table 2.

### 2.4. Evaluation Method

In this study, the single-factor pollution index, Pn, and latent ecological risk index methods were used to evaluate the pollution status of metal contamination in groundwater at the site. Simultaneously, the correlation analysis of metals elements and PCA methods were used to analyze the sources of these metals, which can provide accurate and effective reference information for future site remediation and construction activities [15].

#### 2.4.1. Single-Factor Pollution Index Method

The single-factor pollution index method calculates the worst single index among all indexes to be evaluated. Using the test results of each evaluation factor and the environmental quality classification standard selected for the project, its category was determined, and its attribution category was determined by comparing the test results for the evaluation factor with the classification standard adopted by the project. The single-factor pollution index method can strengthen the objectivity in determining the index weight [16,17]. The single-factor index method was used for evaluation. The pollution indices of the pollutants were calculated by comparing measured values with the evaluation standards. The standard limits of this study were subject to Class IV in Table 1 and Table 3 of the Groundwater Quality Standard GB/T14848-2017 [18].

Formula (1):

(1) $$P_{i}=\frac{C_{i}}{S_{i}}$$

In the formula:

$P_{i}$: pollution index of the *i*th metals.

$C_{i}$: concentration of the element *i*th.

$S_{i}$: evaluation criteria for the *i*th metals.

#### 2.4.2. Nemerow Comprehensive Pollution Index Method

As a pollution evaluation index, the *P_n_* can highlight serious pollution evaluation factors while accounting for the contribution of different factors in the evaluation system while simultaneously reducing the influence of subjective factors in the weighting process. This is a comprehensive evaluation method [19]. Table 3 shows the classification of single-factor and comprehensive pollution indices.

Formula (2):

(2) $$P_{n}=\sqrt{\frac{{\bar{P}}^{2}+P_{max}^{2}}{2}}$$

In the formula:

$P_{n}$: comprehensive pollution index of metals at a sampling point.

$P_{max}$: maximum value of the single-factor pollution index of each metal pollutant at the sampling point.

$\bar{p}$: average value of the single-factor pollution index.

#### 2.4.3. Potential Ecological Hazard Index Method

The potential ecological hazard index was proposed by Hankanson [20]. This method is widely used to evaluate the ecological hazards of metals in environmental media based on the biological toxicity of metals [21]. The calculation method is shown in Formulas (3) and (4).

(3) $$C_{f\;}^{i}=\frac{C_{s}^{i}}{C_{n}^{i}}$$

(4) $$E_{r}^{i}={\;T}_{r}^{i}\times C_{f}^{i}$$

(5) $$RI=\sum_{i=1}^{n}E_{r}^{i},$$

In the formulas:

$C_{f}^{i}$: the pollution coefficient of the *i*th metal.

$C_{s}^{i}$: the actual determined concentration of the element [22].

$C_{n}^{i}$: the reference value of the *i*th metal (μg/L). In this study, the standard metal values in groundwater at the site were used as reference ratios, and these values are listed in Table 4.

$E_{r}^{i}$: the potential hazard coefficient of the *i*th metal.

$T_{r}^{i}$: the toxicity response coefficient of the *i*th metal (the value is shown in Table 3).

*RI*: the potential ecological hazard index of the *i*th metal.

The grading standards of evaluation indicators are $E_{r}^{i}$ < 40, 40 ≤ $E_{r}^{i}$ < 80, 80 ≤ $E_{r}^{i}$ < 160, 160 ≤ $E_{r}^{i}$ < 320, and 320 ≤ $E_{r}^{i}$, which correspond with slight, moderate, strong, very strong, and extremely strong ecological risk degrees of single-factor pollution, respectively, and *RI* < 150, 150 ≤ *RI* < 300, 300 ≤ *RI* < 600, and 600 ≤ *RI*, which correspond with low, moderate, severe, and very severe potential ecological risk degrees, respectively.

#### 2.4.4. Correlation Analysis

Correlation analysis is a statistical method used to quantify the strength and direction of a relationship between two or more variables. It measures this relationship by calculating a correlation coefficient, which typically ranges from −1 to 1. The primary purpose of correlation analysis is to understand the interrelationships between variables and provide a basis for data analysis and decision-making. Here, the Pearson correlation coefficient method is used to quantify the linear relationships between the variables, and its correlation coefficient numerically quantifies the linear relationship between two variables, with a range of −1 to +1. Moreover, +1 indicates a perfect positive correlation (as one variable increases, the other increases proportionally), −1 indicates a perfect negative correlation (as one variable increases, the other decreases proportionally), and 0 indicates no linear relationship between the two variables [23].

By calculating the Pearson correlation coefficient, we can intuitively understand whether there is a linear relationship between the variables and their degrees of strength.

#### 2.4.5. Principal Component Analysis

PCA is a statistical method that simplifies multiple correlated variables into a few uncorrelated composite variables (principal components) using dimensionality reduction techniques while retaining as much of the variance of the original data as possible. The core idea of PCA is to project the data onto new coordinate axes (principal components) through linear transformation, such that these new axes can explain the variance of the data to the greatest extent [24].

The KMO (Kaiser–Meyer–Olkin) test is used to assess the correlation and partial correlation between variables to determine whether the data are suitable for factor analysis or principal component analysis. Generally, a KMO value greater than 0.5 is considered acceptable for principal component analysis. Bartlett’s test of sphericity is used to examine whether there is significant correlation among variables to determine whether the data are suitable for factor analysis. Typically, a p-value less than 0.05 indicates that the data are suitable for factor analysis [25].

To further analyze the pollution sources of metals, PCA was carried out on six metals (Tl was excluded from the analysis because its concentrations were low). However, before adopting this method, it was necessary to determine the appropriateness of using the metals data with KMO (Kaiser–Meyer–Olkin) and Bartlett tests. The KMO test value was 0.577, and the Bartlett sphericity test was <0.001, which proved that this method could be appropriately applied to the data [24].

### 2.5. Data Statistics and Processing

In this study, SPSS (version 27.0) was used for the correlation analysis, and Origin 2021 was used to generate the heat map for the correlation analysis of metals in groundwater and the radar map of metal content.

## 3. Results and Discussion

### 3.1. Characteristics of Metal Content in Site Groundwater

**Figure 2:**
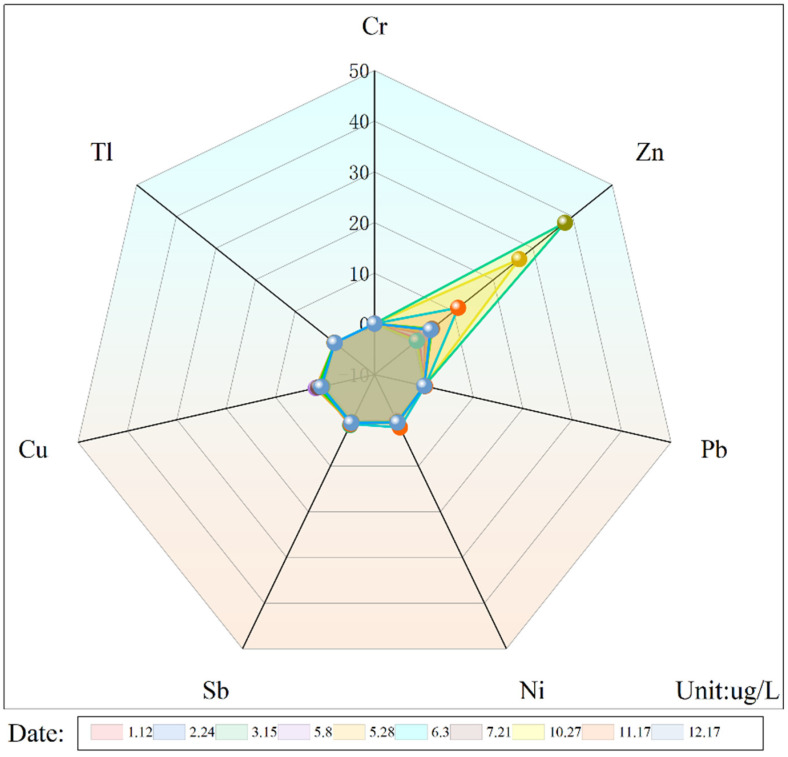
Concentration radar chart of metals in groundwater from a contaminated site in Jiujiang City, China.

The basic physicochemical properties of seven metals in the groundwater at five sampling points of the site were determined. The pH range at the sampling points was between 7.86 and 8.1. As shown in Table 5, the maximum values of the seven metals (Cr, Zn, Pb, Ni, Sb, Cu, and Tl) were lower than the Class IV limit of the groundwater quality standard. The radar chart (Figure 2) shows that the highest measured concentration of any metal at this site was Zn (38.08 µg/L). This environmental concentration was extremely low, making subsequent site remediation relatively easy. The average concentration of heavy metals at each of the five sampling points (*n* = 10) is shown in Table 6.

### 3.2. Evaluation of Metal Pollution in Groundwater

#### 3.2.1. Single-Factor and Nemerow Comprehensive Pollution Index Methods

**Figure 3:**
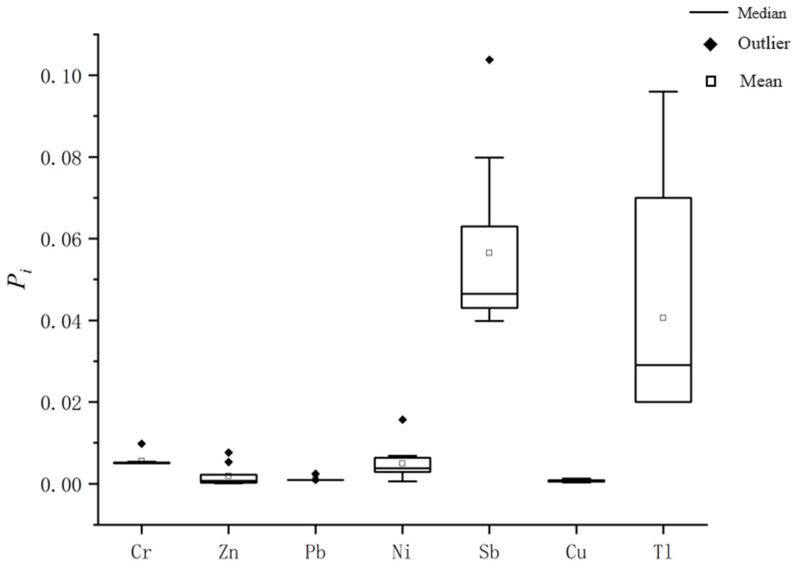
Box plot of the single-factor pollution index (P_i_) of metals in groundwater from a polluted site in Jiujiang City, China.

As shown in Table 7, the intensity of metal pollution decreased in the order of Sb (0.0565) > Tl (0.0406) > Cr (0.00554) > Ni (0.004896) > Zn (0.001862) > Pb (0.001056) > Cu (0.000707). Based on the Pi ≤ 1, all seven metals could be classified at the non-pollution level, indicating that the degree of groundwater by metals at this site was low. Pn was 0.01588 (<0.7) and could be classified as Class I non-pollution. From the box plots of the single-factor pollution index in the study area (Figure 3), it was evident that the Pi values of the seven metals were all lower than 1. Therefore, it can be concluded that the groundwater at this site is negligibly contaminated by these metals.

#### 3.2.2. *Potential Ecological Hazard Index Method in Groundwater*

Based on the evaluation of the potential ecological risk posed by individual metals, the potential ecological risk from metals in groundwater at this site was analyzed. The results showed that the potential ecological risk values of single elements were Tl (0.96) > Sb (0.519) > Pb (0.121) > Ni (0.0783) > Cr (0.01108) > Cu (0.00634) > Zn (0.00186). The average $E_{r}^{i}$ value was <40, and the potential risk level was slight. The potential ecological risk degree (RI) was 1.59, which is classified as low (<150). In summary, the degree of ecological risk from metal pollution in groundwater at this site was very low, with the predicted impacts on the surrounding environment and organisms likely to be small.

### 3.3. Correlation Analysis of Metal Elements in Groundwater

**Figure 4:**
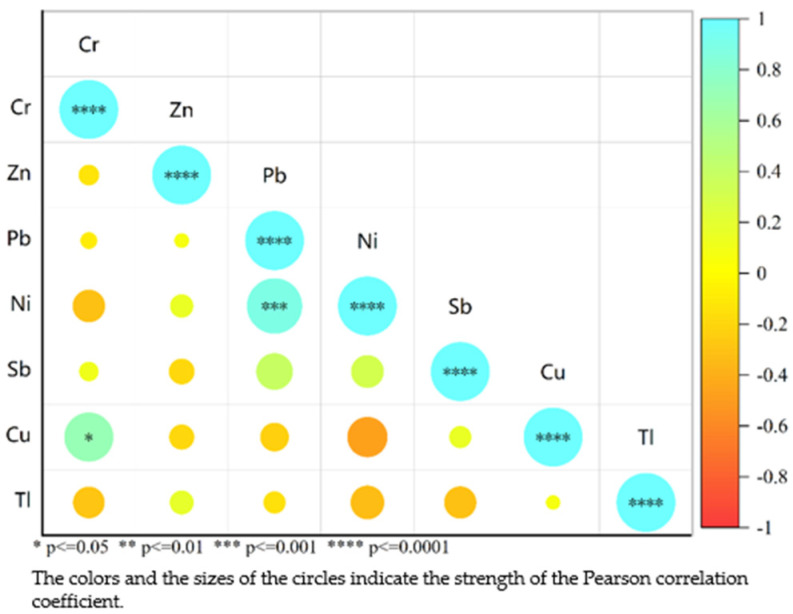
Correlation heatmap of metals in groundwater at a contaminated site in Jiujiang City, China.

The Pearson correlation analysis between metals is shown in the heat map of metal correlations shown in Figure 4. The metals Pb and Ni show a significant positive correlation, indicating that Pb and Ni may have the same source or migration and transformation processes, while the correlation between the metal elements Cr and Cu is positive; however, there is a less significant correlation with Pb and Ni, indicating that Cr and Cu may have the same source or migration and transformation processes [4]. At the site, the potential sources of metals were sewage or wastewater, which came from (1) domestic sewage generated in the site office area, (2) wastewater from soil remediation, (3) vehicle-washing wastewater.

### 3.4. Principal Component Analysis 

**Figure 5:**
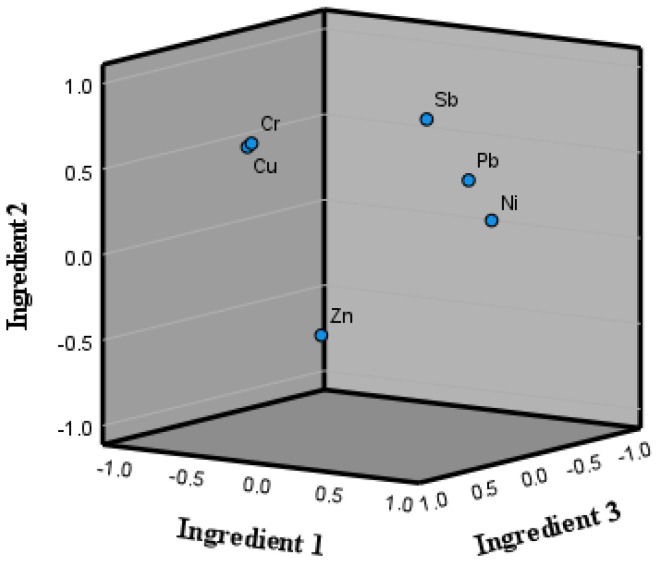
Principal component analysis (PCA) plot of the main principal components of metals in groundwater from a contaminated site in Jiujiang City, China.

Three principal components were extracted, the eigenvalue was greater than 0.967, the contribution rates of principal components 1, 2, and 3 were 40.826%, 27.523%, and 16.130%, respectively, and the cumulative contribution rate was 84.479%, which was greater than 80% (Table 8); thus, most of the variance in the data was explained by the three components. The loading of each metal on the three principal components is shown in Table 9. Both Table 9 and Figure 5 show that Pb, Ni, and Sb were highly associated with component 1. The strong association and distribution of these three metals may be due to the presence of solid waste at the site, such as abandoned structures, construction waste, and waste residue in the plot [26,27,28], as well as human activities such as wastewater from soil remediation and vehicle-washing wastewater. While Cr and Cu were strongly associated with principal component 2, their concentrations were very low.

In summary, the sources of several metals at this site can be derived from the residues of materials accumulated in the raw material warehouse of the former ironmaking plant. Others have little impact on the concentration of metals in the groundwater at this site, and all subsequent restorations at this site will be relatively simple.

### 3.5. Comparison with Heavy Metals in Groundwater from Other Places

Comparisons with heavy metals in groundwater from other places (µg/L) are shown in Table 10. We found that the pollution level in the area that we surveyed was relatively low, and the detected concentrations of Cr, Zn, Pb, Ni, and Cu in groundwater were significantly lower than those in Mathura, India. The reason may be that Mathura’s industrial emissions and the application of fertilizers and sludge to farmland are more severe, which may lead to soil pollution and increase the absorption of heavy metals. Similarly, in areas of Italy with high groundwater levels, agricultural activities may be the main reason for high nickel concentrations. The use of nickel-rich insecticides or fertilizers may be the main reason for the increase in nickel concentration in groundwater samples. It was found that the average concentration of zinc in this study was 12.8 µg/L; this may be caused by urban landfill and agricultural fertilization or by the use of zinc as a fossil fuel additive in car engines, which also pollutes the environment due to exhaust emissions.

## 4. Conclusions

(1) The average concentrations of seven metals (Cr, Zn, Pb, Ni, Sb, Cu, and Tl) detected at this site were all lower than the standard limits in the groundwater quality standard Class IV water standard (GB/T14848-2017).

(2) The Pi calculated using the single-factor pollution index method was <1 for all seven metals (thus, they were classified as causing no pollution), while the comprehensive Pn was 0.01588 (<0.7, which was classified as level I: no pollution).

(3) Using the potential ecological hazard index method, the potential risk values of the seven metals were calculated according to the evaluation of the potential ecological risk grade of the individual metals. The potential risk values of the seven metals ($E_{r}^{i}$) were <40, which is a slight latent load risk level. RI < 150 indicated a low potential risk.

(4) Lead and Ni showed a significant positive correlation, indicating that they may have the same source or migration and transformation processes, while there were positive correlations and negative correlations between other metals, though these were not as significant. Three principal components were extracted through PCA with a cumulative contribution rate of 84.479% when the characteristic root value was greater than 0.967. Lead, Ni, and Sb were strongly loaded on principal component 1. According to their distribution characteristics, the presence of these three metals may likely be due to the presence of solid wastes at the site (such as abandoned structures, construction waste, and waste residue) and wastewater from activities such as soil remediation and vehicle washing.

In summary, the contamination levels of the seven metals in groundwater at the site were very low. Our analysis provides a scientific basis for assessing the degree of groundwater contamination at the site. The results indicate that future site remediation and construction activities at the site do not require a strong focus on metal contamination in groundwater, thereby reducing the workload. Moreover, by combining correlation analysis and PCA to infer the sources of the main pollutants, it is possible to prevent increased metal contamination in groundwater at the site from the source.

## 5. Perspectives

This study only conducted an environmental risk assessment on the concentrations of the seven metals with the highest content among the measured elements in the groundwater of the site; we did not investigate the migration and transformation of these metal elements. It remains uncertain whether the toxicity of the site will increase in the future. Therefore, future research should further develop the monitoring and analysis of the migration and transformation processes of metal elements to more comprehensively assess the potential environmental risks at the site. This will not only help predict the trends in toxicity changes but also provide a scientific basis for the development of more effective pollution control measures [29].

Groundwater metal pollution poses a potential threat to aquatic ecosystems. Conducting risk assessments based on toxicity data from fish, algae, Daphnia, and microorganisms can comprehensively reveal the ecological effects of contaminants [30]. Future research should integrate multi-species toxicity data to construct species sensitivity distribution (SSD) curves, assessing the sensitivity of different biological groups to metallic pollutants. Additionally, the study of metal migration and speciation changes in groundwater–surface water interactions, combined with microbial toxic responses and remediation potential, can shed light on the long-term impacts of pollutants on ecosystems [31]. By quantifying uncertainties and establishing scientific water quality standards, theoretical foundations and practical guidance for the risk management and ecological remediation of groundwater metal pollution can be provided. The integration of interdisciplinary approaches and advanced technologies will drive innovative developments in this field.

## Figures and Tables

**Table 1:** The detection/quantitation limits of the ICP-MS.

Unit: μg/L	Detection Limit	Quantitation Limit
Cr	0.015	0.045
Zn	0.0521	0.156
Pb	0.0186	0.0558
Ni	0.0045	0.0135
Sb	0.00494	0.0148
Cu	0.018	0.054
Tl	0.002	0.006

**Table 2:** Element working curves and correlation coefficients.

Unit: μg/L	Internal Standard Elements and Concentration Range of Multi-Element Standard Solution Used (μg/L)	Correction Curve Equation	Correction Correlation Coefficient r
Cr	Sc (5~100)	y = 0.207x + 0.287	0.9999
Zn	Ge (5~500)	y = 0.036x − 0.058	1.0000
Pb	Bi (5~500)	y = 0.209x + 0.032	1.0000
Ni	Ge (5~500)	y = 0.182x − 0.261	0.9999
Sb	Rh (5~500)	y = 0.002x + 0.006	0.9999
Cu	Ge (5~500)	y = 0.500x + 0.219	1.0000
Tl	Bi (5~500)	y = 0.032x + 0.295	0.9995

**Table 3:** Classification of the single-factor pollution index and Nemerow comprehensive pollution index.

Single-Factor Pollution Index	Grade	Nemerow Comprehensive Pollution Index	Grade
*P_i_* ≤ 1	Class I, no contamination	P Synthetic ≤ 0.7	Class I, no contamination
*P_i_* ∈ (1, 2]	Class II, slightly contaminated	P Synthesis ∈ (0.7, 1.0]	Class II, slightly contaminated
*P_i_* ∈ (2, 3]	Class III, lightly contaminated	P Synthesis ∈ (1.0, 2.0)	Class III, lightly contaminated
*P_i_* ∈ (3, 5]	Class IV, moderately contaminated	P Synthesis ∈ (2.0, 3.0)	Class IV, moderately contaminated
*P_i_* > 5	Class V, heavily polluted	P Overall > 3.0	Class V, heavily polluted

*P_i_*, pollution index of the *i*th metals.

**Table 4:** Background and toxicity response coefficient values for each element.

Element	Cr	Zn	Pb	Ni	Sb	Cu	Tl
Background content $C_{n}^{i}$ (μg/L)	10	5000	100	100	10	1500	1
Toxicity coefficient $T_{r}^{i}$	2	1	5	5	5	5	10

**Table 5:** Characteristics of metals concentrations in groundwater from a contaminated site in Jiujiang City, China.

Metals	Minimum (µg/L)	Maximum (µg/L)	Mean (µg/L) n = 10	Standard Deviation	Coefficient of Variation (%)	Screening Value (µg/L)
Cr	0.05	0.098	0.0554	0.0150	27.08	10
Zn	0.67	38.08	9.312	12.781	137.25	5000
Pb	0.09	0.342	0.1056	0.0479	45.45	100
Ni	0.06	1.566	0.4896	0.431	88.03	100
Sb	0.398	1.038	0.565	0.208	36.81	10
Cu	0.498	1.9	1.061	0.4299	40.53	1500
Tl	0.02	0.096	0.0406	0.0279	68.97	1

n: The average value of data from five sampling points at the same sampling time was n = 1, and a total of 10 samplings were conducted.

**Table 6:** The average concentration of heavy metals at each of the five sampling points (n = 10).

	Cr (µg/L)	Zn (µg/L)	Pb (µg/L)	Ni (µg/L)	Sb (µg/L)	Cu (µg/L)	Tl (µg/L)
GW1	0.051	10.085	0.095	0.484	0.661	1.188	0.049
GW2	0.074	164.656	0.090	0.405	0.523	1.15	0.041
GW3	0.050	14.832	0.101	0.532	0.59	1.0574	0.038
GW4	0.051	11.80	0.151	0.632	0.545	1.106	0.038
GW5	0.051	8.616	0.091	0.395	0.506	0.802	0.037

**Table 7:** Single-factor and comprehensive pollution index of metals in groundwater from a polluted site in Jiujiang City, China.

Metal Elements	Single-Factor Contamination Index Range	Single-Factor Pollution Index Average	Pollution Index	Comprehensive Pollution Index	Contamination Level
Cr	0.05–0.0098	0.00554	No pollution	0.01588	No pollution
Zn	0.000134–0.007616	0.001862	No pollution		
Pb	0.0009–0.00242	0.001056	No pollution		
Ni	0.0006–0.01566	0.004896	No pollution		
Sb	0.0398–0.1038	0.0565	No pollution		
Cu	0.000332–0.001267	0.000707	No pollution		
Tl	0.02–0.096	0.0406	No pollution		

**Table 8:** Total variance explained by principal component analysis.

Principal Component	Initial Eigenvalues			Sum of Squared Loadings for Extraction		
	Sum	Percentage of Variance	Accumulation %	Total	Percentage of Variance	Accumulation %
1	2.450	40.826	40.826	2.450	40.826	40.826
2	1.651	27.523	68.349	1.651	27.523	68.349
3	0.968	16.130	84.479	0.968	16.130	84.479
4	0.586	9.762	94.241			
5	0.266	4.441	98.681			
6	0.079	1.319	100.000			

**Table 9:** Loading of metals with principal components.

Metals	Component 1	Component 2	Component 3
Cr	−0.019	0.913	−0.004
Zn	0.058	−0.050	0.951
Pb	0.934	−0.104	0.082
Ni	0.880	−0.356	0.151
Sb	0.650	0.258	−0.408
Cu	−0.171	0.898	−0.133

**Table 10:** Comparison with heavy metals in groundwater from other places (µg/L).

Place	Cr	Zn	Pb	Ni	Cu	Tl
This study	0.0554	9.31196	0.1056	0.4896	0.565	1.0606
Mathura, India [29]	1900	105	2290	3373	400	/
Guangzhou [30]	3.3	27.99	1.61	/	1.87	/
Lingjiang, Zhejiang [31]	7.21	37	4.45	1.91	4.00	/
Campania Plain, Southern Italy [32]		12.8	1.22	3.66	3.87	

## Data Availability

The data that support the findings of this study are available from the corresponding author upon reasonable request.
